# A Novel Read Scheme for Large Size One-Resistor Resistive Random Access Memory Array

**DOI:** 10.1038/srep42375

**Published:** 2017-02-10

**Authors:** Mohammed Zackriya, Harish M. Kittur, Albert Chin

**Affiliations:** 1 Department of Electronics Engineering, National Chiao Tung University, Hsinchu 300, Taiwan; 2 School of Electronics Engineering, VIT University, Vellore, India

## Abstract

The major issue of RRAM is the uneven sneak path that limits the array size. For the first time record large One-Resistor (1R) RRAM array of 128x128 is realized, and the array cells at the worst case still have good Low-/High-Resistive State (LRS/HRS) current difference of 378 nA/16 nA, even without using the selector device. This array has extremely low read current of 9.7 μA due to both low-current RRAM device and circuit interaction, where a novel and simple scheme of a reference point by half selected cell and a differential amplifier (DA) were implemented in the circuit design.

The RRAM implemented in crosspoint array structure promises high scalability and 3D architecture. A RRAM cell is placed in each intersecting point of Word-Line (WL) and Bit-Line (BL) as shown in Fig. 1(a). During write phase, the RRAM cell is programmed to HRS (R_off_) or LRS (R_on_) to store 0 or 1. To read the resistive state of a RRAM cell, a potential difference of a read voltage (V_read_) is applied to the cell and is termed as Full Selected Cell (FSC). The remaining Non-Selected Cells (NSCs) in the array are biased at 0 potential difference between their terminals. Among various biasing schemes for NSCs, the V_read_/2 method has lower voltage swing and low power consumption. As shown in Fig. 1(a), to read the state of selected cell at 1 × 3, WL1 is pulled to V_read_ while the BL3 is grounded. Rest of the WLs and BLs are biased atV_read_/2. The current passing through the R_sense_ determines the state of the selected RRAM cell. Here the NSCs with one terminal connected to either WL1 or BL3, has potential difference of V_read_/2 between their terminals. These cells are defined as Half Selected Cells (HSC). These HSCs create sneak path to influence the current passing through R_sense_. As shown in a simple 2 × 2 RRAM array of Fig. 1(b), although a RRAM cell at 1 × 2 (green color) is selected; other paths (grey color) through the HSCs can direct current to R_sense_ and disturb the actual current flowing through the selected cell. Thus effective resistance (R_eff_) which is calculated w.r.t. current flowing through the selected BL and V_read_ applied at the WL gets disturbed. The sneak current is much more severe as the array size increases and it is data pattern dependent. The worst case for usual crosspoint array is when the read cell is at HRS while all other cells are at LRS or vice versa^1^,^2^,^3^,^4^,^5^,^6^.

To address the sneak path issue, the RRAM devices (1 R) were proposed in series with a diode (1D1R), a transistor (1T1R) and a selector (1S1R)^1^,^2^,^3^,^4^. Other works have been presented which aim on architectural design (device + circuit interaction) to effectively detect the state of RRAM cell in the presence of sneak current^5^,^6^. In this paper, we present a novel architecture: record large 1 R RRAM array of 128 × 128 is realized with ultra-low read current of 9.7 μA by this scheme, even without selector device. This is possible because the sneak path current is used as reference input for the DA, and the current change of a selected cell from HSC to FSC is much higher at LRS than HRS to differentiate the stored 1 or 0 state.

## Methods

**Figure 2:**
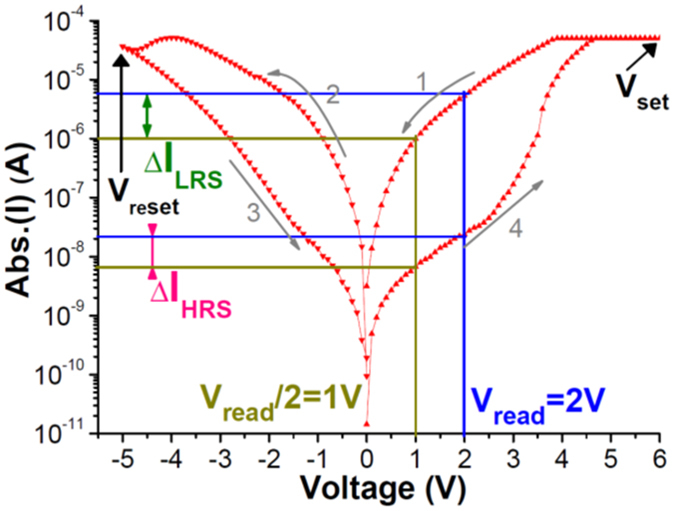
*I-V* hysteresis curves of Ni/SiO_X_/TaN RRAM. The large HRS/LRS ratio is the merit for this device even without selector.

To reach the low set/reset power, we pioneered the covalent-bond-dielectric GeO_x_ RRAM device^7^,^8^. Very low HRS/LRS currents were achieved by carrier hopping conduction rather than the high current via shorted metallic filament. To further control the process-induced resistance variation, similar covalent-bonded SiO_x_ RRAM device was used in this study. Figure 2 shows the *I-V* hysteresis curves of Ni/SiO_x_/TaN RRAM device. This RRAM cell offers large HRS/LRS ratio that is crucial to reach a large size memory array. Besides, the low LRS current allows low power read operation. Table 1 comprises of RRAM device parameters. External reference circuit becomes impractical because of device to device resistance variation. To consider this issue, we have taken lowest HRS and highest LRS to realize the read scheme. Here V_read_ was chosen carefully, as high V_read_ will increase the sneak current. The HRS of the device biased at V_read_ and V_read_/2 are 83.4 and 149.4 MΩ respectively. Similarly the LRS of the device biased at V_read_ and V_read_/2 are 0.38 and 1.0 MΩ respectively. Such high resistance values are crucial to save the read power in a large RRAM array. These parameters were used for simulation to realize an effective read scheme. The proposed architecture determines the state of RRAM cell in an array by considering the current through HSCs and current slope of the FSC. Thus the worst case for the proposed architecture is, when the FSC (last bit of first word) is of HRS (or LRS) and remaining bits are also at HRS (or LRS). The worst case data pattern for the FSC to be HRS and LRS are defined as WC_H_ and WC_L_, respectively. The impact of sneak path increases with the array size and has more impact on WC_L_than WC_H_. This is because WC_L_ has all the cells at LRS in HSC state (LRS_HSC_); thus, these LRS_HSCs_ leak more current through sneak path via resistive network to the selected BL.

## Results

### Background Analysis for Proposed Design

**Figure 3:**
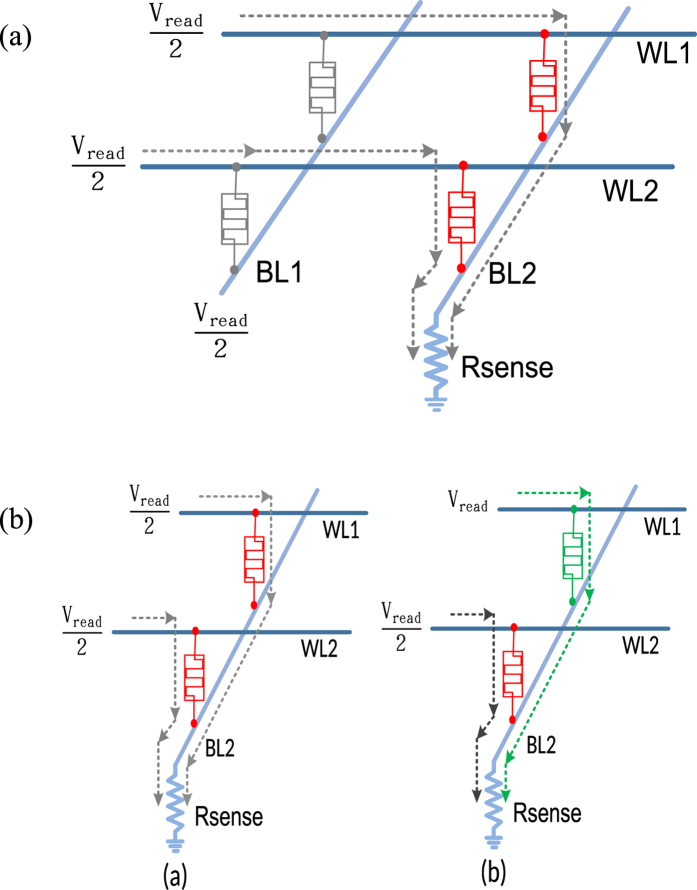
(**a**) 3D structure of 2 × 2 RRAM crosspoint array – cell to be read is located in BL2 and thus it is grounded to measure the leakage current in BL2, and (**b**) Partial 3D structure of 2 × 2 RRAM crosspoint array – Green cell is to be read. (**a**) BL2 is grounded; (**b**) BL and WL are selected (WL1 to V_read_ and BL2 to 0 V).

While reading a particular RRAM cell in the crosspoint array, its corresponding WL and BL are assigned to V_read_ and ground respectively. Initially, both WL and BL were at V_read_/2 potential. As shown in Fig. 3(a), when a cell in BL2 of a 2 × 2 RRAM array is to be read, the BL2 goes to ground. All the cells in BL2 are HSCs (red) and rest of the cells in other BLs are NSCs (grey). The current flowing through R_sense_ is purely the contribution made by the HSCs of the BL2. When the WL1 is assigned to V_read_ from V_read_/2, only the selected cell (green) on BL2 goes from HSC to FSC as shown in Fig. 3(b). When the selected cell moves from HSC to FSC state, the cell resistance reduces and the current through the cell increases. Yet, extra leakage current flows from WL2 to BL2 (dark grey), as it carries leakage current from HSC_WL_ as well as HSC_BL_.

**Figure 4:**
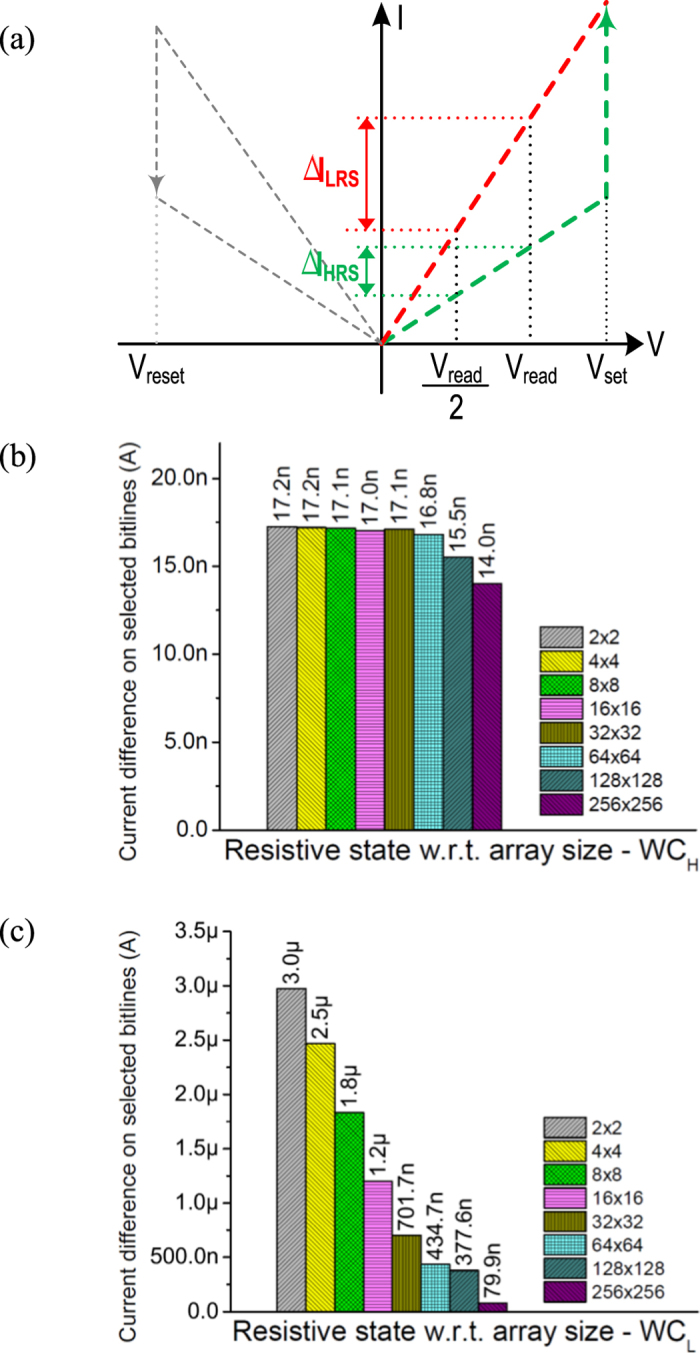
(**a**) Generic switching behavior of RRAM – swept *I-V* curves. HRS and LRS curves are in green and red colors resp., (**b**) Difference in current on selected bitline when the corresponding selected wordline is at V_read_ and V_read_/2 for worst-case data pattern, **WC**_**H**_, and (**c**) Difference in current on selected bitline when the corresponding selected wordline is at V_read_ and V_read_/2 for worst-case data pattern, **WC**_**L**_.

As shown in Fig. 4(a), the current difference on *I-V curves* of RRAM between V_read_/2 and V_read_ is much higher for LRS than HRS. Thus, when the selected cell changes from HSC to FSC, the change in current is very high if the selected cell is LRS compared to HRS. It is further shown in Fig. 4(b,c), the plot of current difference when the WL1 is selected and moving from V_read_/2 to V_read_ for WC_H_ and WC_L_ respectively. Even as the array size increases, WC_H_ remains less than WC_L_. For the best case, the current difference is further less and more while reading HRS (<WC_H_) and LRS (>WC_L_) cell respectively. It can be noticed that, as the array size increases, the difference in current is falling down. This is because of sneak current offered by WL. The DA design^9^ can detect ΔV_in_ as low as 10 mV with an offset of about 5~8 mV. Thus, with R_sense_ of 100 KΩ, the DA can detect the state of 1 R RRAM cell in an array as large as 128 × 128 (voltage difference at input of DA for WC_L_ is 43.5 mV). Beyond 128 × 128 array size, the detectable voltage difference goes below 10 mV. Here the read current for this sized array is only 9.7 μA. The 128 × 128 array size is the largest among 1 R RRAM arrays and proposed architecture is the only architecture available which is close to 1S1R RRAM arrays. This is the basic concept leading to use the reference point by HSC and a DA for circuit design.

### Architectural Implementation

**Figure 5:**
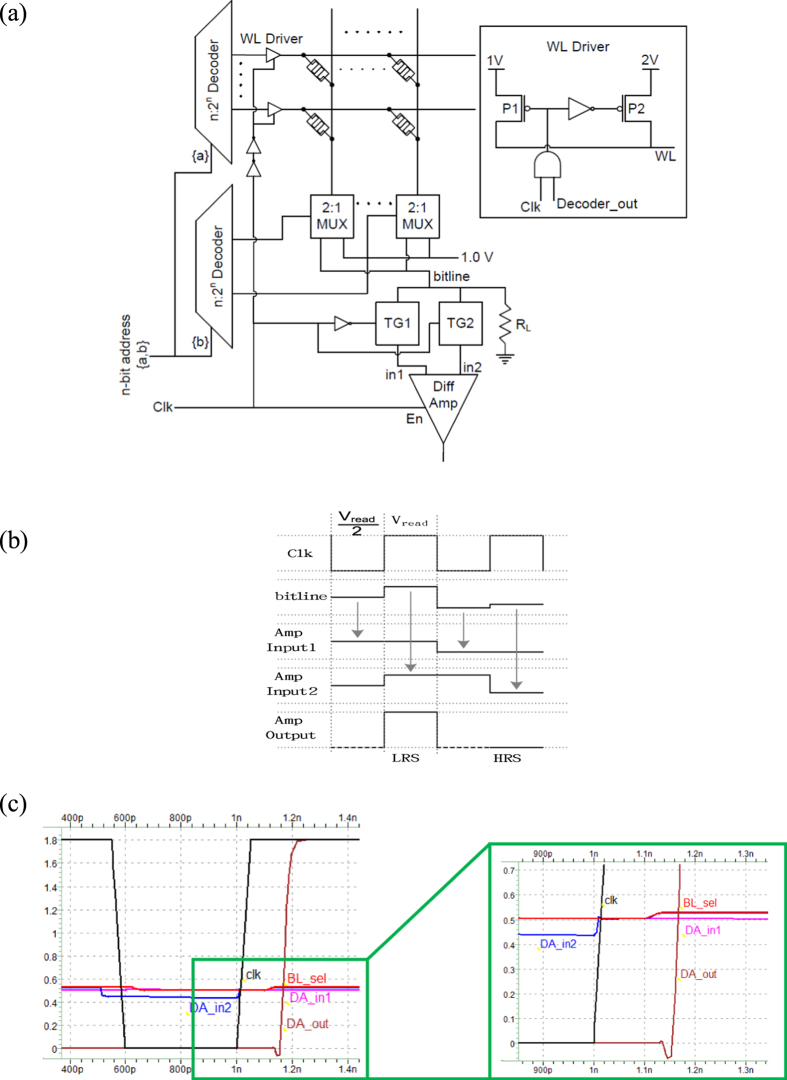
(**a**) Proposed architecture for novel read scheme - a nxn RRAM crosspoint array with differential amplifier. TG – Transmission Gate | MUX – Multiplexer, (**b**) Ideal timing waveform of the proposed read scheme, and (**c**) The simulated waveform of the proposed read scheme for worst case data pattern, WC_L_.

A RRAM crosspoint array architecture synchronized with a clock is presented in Fig. 5(a). Input address drives the row and column decoder to select the WL driver and MUX. Once a MUX is selected, the corresponding BL is grounded through R_sense_. While the WL driver, Transmission Gate (TG) and DA are controlled by the clock. The inset of Fig. 5(a) shows the WL driver circuit, selected WL is held at 2 V (V_read_) when clock is at level-1 and at 1 V (V_read_/2) rest of the time. When the clock is low (to read the cell at 1 × 2), WL driver holds the WL1 at V_read_/2 and through TG1, in1 of DA is charged to the potential of BL2. Similarly, when clock is high, WL driver drives WL1 to V_read_ and through TG2, in2 of DA is charged to the potential of BL2 (while TG1 isolates in1 from BL2, thus in1 replicates the charge on BL2 when WL1 was V_read_/2). The DA is enabled to positive level of the clock to determine the state of selected cell. The operation is further explained in Fig. 5(b); when the clock is low, sneak current through the HSCs (biased at V_read_/2) in BL is captured at the input1 (reference) of DA. As the clock goes high, the potential difference across selected cell becomes V_read_ and the corresponding current is captured on the other input of the DA to determine the resistive state. Buffer is placed between clock and driver, so that clock has sufficient time to turn on and off the TG2 and TG1 respectively, before the driver sets a bias voltage of V_read_ on SC. Simulation result is shown in Fig. 5(c); the DA output goes high as the clock becomes high to indicate the SC is at LRS. The enlarged version of waveform is presented in the inset of Fig. 5(c), in1 of DA follows BL (SC is biased at V_read_/2) when the clock is low. As soon as the clock goes high, SC is still biased at V_read_/2 to avoid disturbance on in1 and thus in1, in2 and BL remains at same potential. After a delay of buffer, the driver sets bias of V_read_ on SC, at this moment in2 of DA follows BL and the DA detects the state of SC. The simulation was performed in HSPICE using TSMC 0.18 μm technology, while modeling and programming was with MATLAB. As presented in Table 2, the design has considered both capacitor and resistor of interconnects during simulation. With the proposed read scheme and low switching power RRAM cell, the stored state of RRAM cell in a 128 × 128 array can be effectively determined even without the selector. This is the largest size among any array presented in literature for 1 R RRAM^1^,^2^,^3^,^6^, even close to among those with 1S1R devices^1^,^2^,^3^. Besides, the 1S1R device has low resistance in the range of KΩ at LRS, which consumes ~3 orders of magnitude more power than this 1 R device in MΩ range. These devices also consume very low power compared to DRAM; yet the speed is 2~3 orders of magnitude slower.

## Conclusion

A novel read scheme is proposed that effectively reads a 1 R cell in an array of 128 × 128. The scheme determines the state of RRAM cell from the difference of the current when the selected WL is at V_read_/2 and V_read_. This scheme predicts the leakage current through the HSCs in BL and further extending to predict leakage through both BL and WL.

## Additional Information

**How to cite this article:** Zackriya, M. *et al*. A Novel Read Scheme for Large Size One-Resistor Resistive Random Access Memory Array. *Sci. Rep.* **7**, 42375; doi: 10.1038/srep42375 (2017).

**Publisher's note:** Springer Nature remains neutral with regard to jurisdictional claims in published maps and institutional affiliations.

## Supplementary Material

Supplementary Information

## Figures and Tables

**Figure 1:**
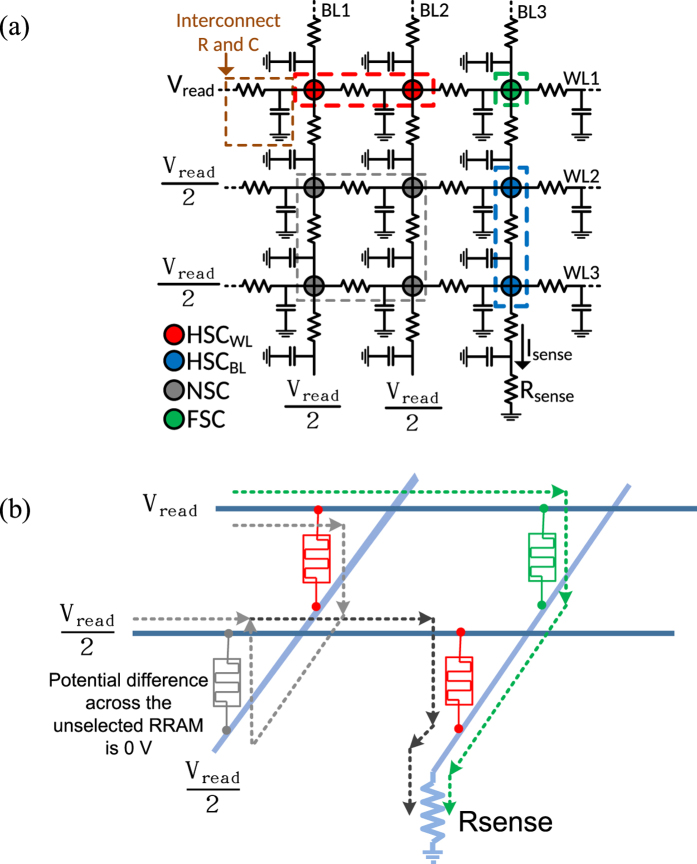
(**a**) 2D structure of RRAM crosspoint array. Rsense– Sense Resistor; FSC– Full Selected Cell; NSC– Non Selected Cell; HSC_WL/BL_– Half Selected Cell WL/BL, (**b**) 3D structure of 2 × 2 RRAM crosspoint array. Green, red & grey cells are FSC, HSC and NSC resp. Actual and leakage current– green & grey path resp.

**Table 1:** Device parameters used for read operation.

Parameters	Values
HRS (R_off_) @ V_read_	83.4 M Ω
LRS (R_on_) @ V_read_	0.38 MΩ
HRS (R_off_)@ V_read_/2	149.4 MΩ
LRS (R_on_) @ V_read_/2	1.0 MΩ
Sense resistor (R_sense_)	100 KΩ
Wire resistor (R_wire_)	2.8 Ω
Wire capacitor (C_wire_)	0.046 fF
Read Voltage (V_read_)	2 V

**Table 2:** Summary of various read scheme of RRAM crosspoint array.

	RRAM Device	Bias Scheme	Array Size	HRS/LRS (MΩ)	V_read_ (V)	Interconnect R/C
J. Zhou *et al*.^1^	1S1R	0 V	128 × 128	1/0.01	1	yes/no
Y. Deng *et al*.^2^	1S1R	V_read_/2	128 × 128	66.6/0.002	2	yes/no
S. H. Jo *et al*.^3^	1S1R	V_read_/2	64 × 64	ratio>10^2^	—	–/–
C. Liu & H. Li^6^	1R	0 V	64 × 64	1/0.005	0.5	yes/no
This work	1R	V_read_/2	128 × 128	83.4/0.38	2	yes/yes
