# Supplementary material for: A Novel Read Scheme for Large Size One-Resistor Resistive Random Access Memory Array
Source: Sci Rep. 2017 Feb 10;7:42375. doi: 10.1038/srep42375 (PMC5301214; doi:10.1038/srep42375)
Supplement: Supplementary Information [file srep42375-s1.pdf]

A Novel Read Scheme for Large Size One-Resistor Resistive Random Access Memory Array

MohammedZackriya V[1,2], Harish M Kittur[2], and Albert Chin[1]

[1]Department of Electronics Engineering, National Chiao Tung University, Hsinchu 300, Taiwan

[2]School of Electronics Engineering, VIT University, Vellore, India

Figure S1(a)-(b), and Figure S1(c)-(d), show the voltage on R_{sense} when the WL1 is at $V_{read}/2$ and V_{read} respectively. Both the worst case patterns, WC_H and WC_L are presented. In case of WC_H, the difference between graphs on Figure S1(a) and S1(b), for 2x2 and 256x256, are 1.7 mV and 1.4 mV respectively. In case of WC_L (Figure S1(c) and S1(d)), for 2x2 and 256x256, are 297.3 mV and 8.0 mV respectively. Thus the proposed architecture is effective till array size of 128x128 which generates voltage difference (at input of amplifier) of 1.55 mV and 43.5 mV for WC_H and WC_L respectively (which is well inside margin of amplifier sensitivity). Even as the array size increases, WC_H remains less than WC_L. For the best case, the potential difference is further less and more while reading HRS ($<WC_H$) and LRS ($>WC_L$) cell respectively.

Supplementary Figures

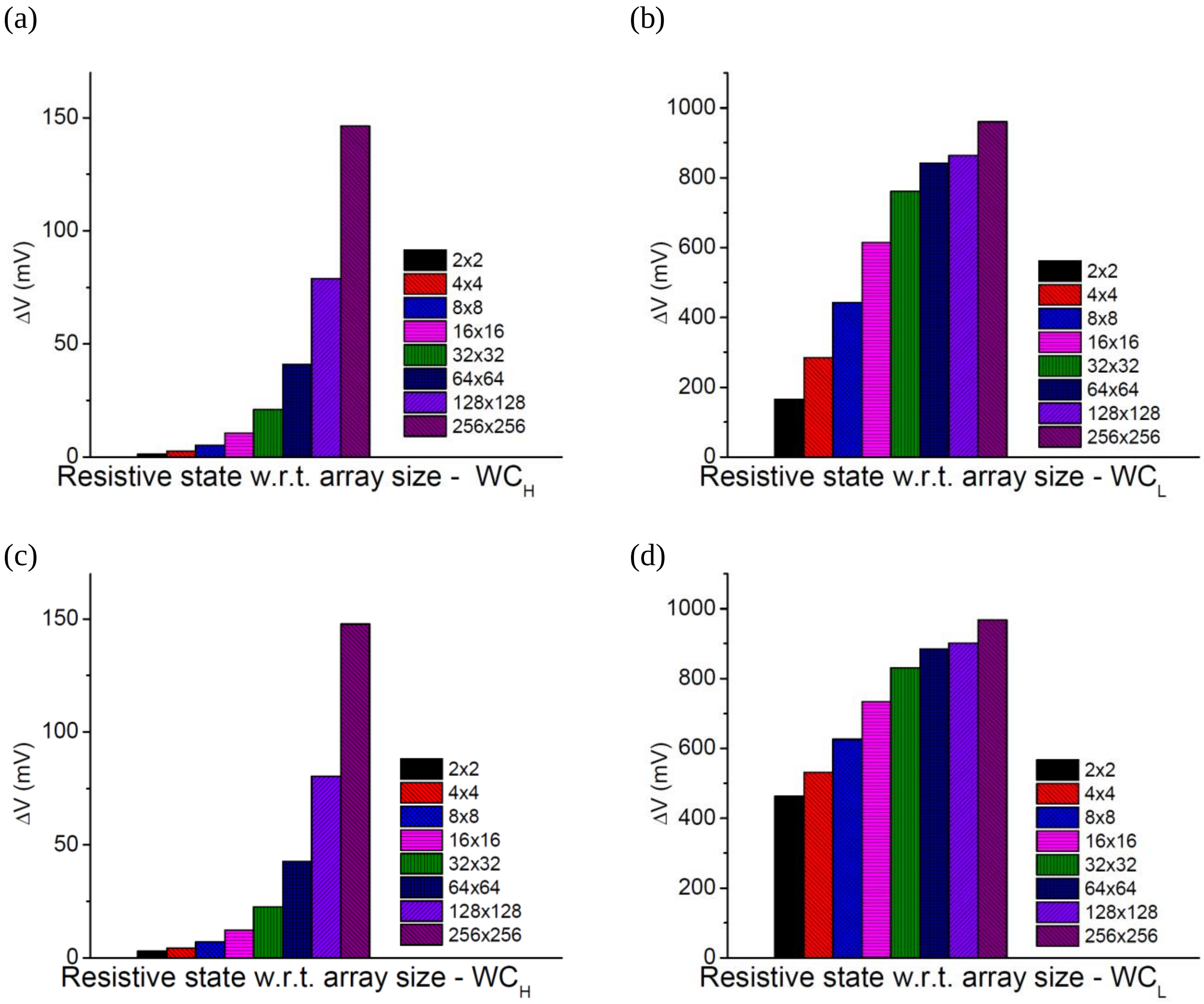

Figure S1. (a) Voltage swing on R_{sense} when just BL is selected (grounded) for worst-case data pattern, $\mathbf{WC_H}$. Selected cell is biased at $\mathbf{V_{read}/2}$, (b)Voltage swing on R_{sense} when just BL is selected (grounded) for worst-case data pattern, $\mathbf{WC_L}$. Selected cell is biased at $\mathbf{V_{read}/2}$, (c) Voltage swing on R_{sense} when both WL and BL are selected ($V_{read}$and ground resp.) for worst-case data pattern, $\mathbf{WC_H}$. Selected cell is biased at $\mathbf{V_{read}}$, (d) Voltage swing on R_{sense} when both WL and BL are selected ($V_{read}$and ground resp.) for worst-case data pattern, $\mathbf{WC_L}$. Selected cell is biased at $\mathbf{V_{read}}$.
